# Cerebrospinal Fluid Beta‐Amyloid Concentration and Clinical and Radiographic Manifestations of Cerebral Amyloid Angiopathy

**DOI:** 10.1161/JAHA.124.040025

**Published:** 2025-03-03

**Authors:** Baris Alten, Elif Gokcal, Andrew Warren, Susanne J. van Veluw, Mariel Kozberg, M. Edip Gurol, Anand Viswanathan, Steven M. Greenberg

**Affiliations:** ^1^ Mass General Brigham Neurology Residency Program Boston MA USA; ^2^ Department of Neurology Massachusetts General Hospital Boston MA USA; ^3^ Department of Neurology Brigham and Women’s Hospital Boston MA USA

**Keywords:** amyloid, cerebral amyloid angiopathy, cerebrospinal fluid biomarkers, cortical superficial siderosis, perivascular spaces in centrum semiovale, intracerebral hemorrhage, tau, Biomarkers, Cerebrovascular Disease/Stroke, Cognitive Impairment, Intracranial Hemorrhage, Magnetic Resonance Imaging (MRI)

## Abstract

**Background:**

Cerebral amyloid angiopathy (CAA) is driven by vascular Aβ (amyloid‐beta) deposition, which can be detected as reduced Aβ species in cerebrospinal fluid (CSF). We sought to identify relationships between CSF Aβ and tau concentrations and various manifestations of CAA.

**Methods:**

This is a retrospective cross‐sectional single‐center study of patients diagnosed with CAA per Boston Criteria version 2.0, had magnetic resonance imaging brain scans, and underwent CSF testing for Aβ and tau concentrations between 2008 and 2022. Associations between clinical/magnetic resonance imaging features and CSF biomarker concentrations were investigated with univariate and multivariate models.

**Results:**

We identified 31 patients aged 69.6±8.4 years, of whom 20 presented with cognitive complaints, 9 with CAA‐related macrohemorrhage (lobar intraparenchymal or convexity subarachnoid hemorrhage), and 2 with transient focal neurological episodes. Presence of macrohemorrhage (301.8±112 pg/mL versus 400.9±123 pg/mL, *P*=0.029), cortical superficial siderosis (309.6±131 mg/dL versus 412.3±100 pg/mL, *P*=0.021), and severe enlarged perivascular spaces in centrum semiovale (285.8±91 pg/mL versus 428.3±117 pg/mL, *P*<0.001) were associated with lower Aβ42 concentrations. Aβ42 concentrations inversely correlated with the number of these manifestations, being lowest in patients having all three. Patients with cognitive complaints had higher t‐tau (total tau; 551±320 pg/mL versus 317.2±141 pg/mL, *P*=0.03) and trended toward having higher p‐tau (phosphorylated tau at threonine 181) concentrations (75.69±39 pg/mL versus 49.24±22 pg/mL, *P*=0.05).

**Conclusions:**

Lower CSF Aβ42, suggesting higher amyloid burden, is associated with CAA‐related macrohemorrhages and severe enlarged perivascular spaces in centrum semiovale, suggesting potential mechanistic links and CSF Aβ42 as a potential biomarker for progression of CAA. CSF tau concentrations are linked to cognitive complaints, likely representing comorbid Alzheimer disease pathology.

**Nonstandard Abbreviations and Acronyms**
Aβ42: amyloid‐beta 1‐42 peptide
CAA: cerebral amyloid angiopathy
cSS: cortical superficial siderosis
CMB: cerebral microbleeds
EPVS‐CSO: enlarged perivascular spaces in centrum semiovale
ICH: intracranial hemorrhage
p‐tau: phosphorylated tau at threonine 181
TFNEs: transient focal neurological episodes
t‐tau: total tau
WMH: white matter hyperintensities

**Research Perspective**

**What Is New?**

- This is the first study to systematically investigate Aβ42 (amyloid‐beta 42) and tau concentrations in cerebrospinal fluid (CSF) in relation to specific clinical and radiographic manifestations of cerebral amyloid angiopathy (CAA), showing lower CSF Aβ42 concentrations with presence of CAA‐related macrobleeds, cortical superficial siderosis, and severe enlarged perivascular spaces in centrum semiovale and higher tau concentrations with presence of cognitive complaints.
- The findings raise the possibility that macrobleeds, cortical superficial siderosis, and severe enlarged perivascular spaces in centrum semiovale may be aspects of CAA that specifically associate with total burden of vascular Aβ as estimated by reduced CSF Aβ42 concentrations.
- Elevated CSF tau concentrations in patients with CAA presenting with cognitive complaints signify neurodegeneration, reflecting either the impact of vascular amyloid deposition on parenchymal damage or comorbid Alzheimer disease pathology.

**What Question Should Be Addressed Next?**

- What is the mechanistic link between higher vascular amyloid burden and the development of CAA‐related macrohemorrhages‐a leading cause of intracranial hemorrhage in older adults with very high mortality and morbidity, and how might understanding this link inform the development of targeted diagnostic and therapeutic strategies?
- How do CSF Aβ42 levels change over time, and could these dynamics be used to track specific aspects of disease progression or assess the effectiveness of therapeutic interventions aimed at reducing vascular amyloid burden in CAA?

Cerebral amyloid angiopathy (CAA) is a common cerebral small vessel disease of older adults, which manifests clinically with spontaneous intracranial hemorrhage (ICH), cognitive impairment, or transient focal neurological episodes. Histopathologically, it is characterized by deposition of Aβ (amyloid‐beta) peptide within the walls of the cortical and leptomeningeal vessels.^1^ Although the exact mechanism of widespread brain injury stemming from the vascular amyloid deposition remains unclear, a recently proposed framework suggests a stepwise progression from vascular amyloid deposition to impaired vascular physiology resulting in nonhemorrhagic followed by hemorrhagic manifestations of CAA.^2^

Because vascular amyloid deposition is implicated as the primary cause of the disease, understanding the role of this deposition on disease progression and severity is crucial. Vascular amyloid deposition is associated with reduction in concentrations of Aβ species in cerebrospinal fluid (CSF), which has been reported for both sporadic CAA^3^, ^4^, ^5^, ^6^ and hereditary CAA.^3^, ^7^ For example, in a population‐based autopsy study, individuals with pathologically confirmed CAA were found to have lower CSF Aβ concentrations compared with those without; and higher vascular amyloid burden was associated with lower CSF Aβ concentrations among those with CAA.^5^ In carriers of Dutch‐type hereditary CAA, the reduction in CSF Aβ concentrations can be detected as young as in their mid‐20s, approximately 30 years before the mean age of symptomatic ICH, suggesting that lower CSF Aβ concentrations can reliably reflect vascular amyloid deposition even at the earlier stages of the disease process.^2^ Greater amyloid positron emission tomography (PET) signal using Pittsburgh Compound B was also found to be associated with reduced CSF Aβ concentrations in carriers of hereditary CAA, further supporting the association between lower CSF Aβ concentrations and higher amyloid deposition.^8^ Despite our understanding of the strong association between lower CSF Aβ concentrations and pathologically and radiographically shown higher amyloid burden, the relationship between the CSF Aβ concentrations with specific clinical and radiological manifestations of CAA has not been studied to date.

Based on the proposed pathophysiology of continuing amyloid deposition leading to clinical and radiographic manifestations of CAA in a stepwise fashion (abnormal vascular reactivity, followed by nonhemorrhagic and then by hemorrhagic manifestations), we hypothesized that lower CSF Aβ42 (amyloid‐beta 42) concentrations, likely reflecting higher amyloid burden, might be associated with more extensive brain injury in CAA. In addition, we aimed to investigate the associations between various manifestations of CAA and CSF concentrations of amyloid‐β 1–42 peptide (Aβ42), t‐tau (total tau), and p‐tau (phosphorylated‐tau at threonine 181).

## METHODS

Deidentified data supporting the findings of this study are available from Baris Alten upon reasonable request.

### Study Participants

Study subjects were identified retrospectively. We identified participants in this study from 2 sources: (1) search of Massachusetts General Hospital (Boston, MA) electronic medical records and (2) systematic review of the patient panels of the authors' inpatient and outpatient stroke care for individuals carrying a diagnosis of CAA (*International Classification of Diseases, Tenth Revision* [*ICD‐10*] code 168.0) who had undergone lumbar puncture as part of their clinical evaluation for CSF t‐tau/p‐tau/Aβ42 panel via Athena ADMark assay between 2008 and 2022. Analyzed subjects were required to have a brain magnetic resonance imaging (MRI) as part of their clinical evaluation that included a hemosiderin‐sensitive T2*‐weighted susceptibility sequence. A total of 52 unique patients were identified. Out of 52, 21 were excluded because of unavailability of CSF results (8), unavailability of adequate MRI images (1), or not meeting diagnostic criteria for CAA per Boston Criteria version 2.0 (12) (see Figure S1). The remaining 31 consecutive patients meeting eligibility criteria were included in the final analyses. Based on the clinical data and MRI findings, patients were categorized into probable CAA with supporting pathology, probable CAA, or possible CAA according to the Boston Criteria version 2.0.^9^ Demographics were extracted from the medical charts. Cognitive complaints were ascertained by review of outpatient neurology or primary care notes.

### Standard Protocol Approvals, Registrations, and Patient Consent

This study was performed with the approval of and in accordance with the guidelines of the institutional review board of Massachusetts General Hospital with waived informed consent (based on minimal patient risk and practical inability to perform the study without waiver). Strengthening the Reporting of Observational Studies in Epidemiology reporting guideline is used and reported separately.

### Structural MRI

All patients underwent a structural brain MRI that included at least a T2‐weighted sequence, fluid‐attenuated inversion recovery sequence and a T2*‐weighted susceptibility sequence (susceptibility weighted imaging/susceptibility weighted angiography or gradient echo) as part of their clinical evaluation. MRI scans of patients were evaluated by an investigator blinded to demographics and CSF markers. Established markers of CAA were identified and scored, using appropriate MRI sequences. CAA‐related macrobleeds and cerebral microbleeds (CMBs) were identified and quantified, as previously described.^10^ The presence of cortical superficial siderosis (cSS) was assessed based on previously published criteria.^11^ According to these criteria, cSS is defined as a linear hypointensity over the cortex that follows the curvilinear shape of the surrounding cerebral gyri on T2*‐weighted susceptibility sequences. Lacunes were identified according to the Standards for Reporting Vascular Changes on Neuroimaging criteria.^12^ Briefly, lacunes are defined as round or ovoid lesions with a diameter between 3 mm and 15 mm, hyperintense on T2‐weighted images, hypointense on T1‐weighted images, and hypointense on fluid‐attenuated inversion recovery images with a surrounding rim of hyperintensity. The location of lacunes was recorded as deep if the lesion was located in the thalamus, basal ganglia, internal capsule, external capsule, brainstem, or deep cerebellar regions or lobar if the lesion was located in cortical or subcortical nondeep brain regions including centrum semiovale (CSO), frontal, parietal, insular/subinsular, temporal, or occipital lobes.^13^ Enlarged perivascular spaces in the CSO (EPVS‐CSO) were assessed as previously defined and rated with a validated visual rating scale (0=no EPVS, 1=<10 EPVS, 2=11–20 EPVS, 3=21–40 EPVS, and 4=>40 EPVS).^14^ Severe EPVS‐CSO was defined as having a rating scale of 3 and 4 and nonsevere as a rating scale of 0, 1, and 2. The burden of white matter hyperintensities (WMH) was evaluated using the Fazekas grading.^15^ The degree of WMH was classified as high degree when the Fazekas grade was >1 and a low degree when the Fazekas grade was 0 or 1.

### CSF Analyses

CSF samples obtained as part of clinical evaluation were assayed using the commercial ADMark CSF test (Athena Diagnostics), an electrochemiluminescent immunoassay that measures Aβ42, t‐tau, and p‐tau concentrations, which has been validated pursuant to the Clinical Laboratory Improvement Amendments regulations. Quantitative data rely on creating a standard curve using purified reactants of a known concentration that are run in parallel with the test samples; thus, results are comparable among different runs/batches of the same test. In addition, this test provides constant reference ranges for Aβ42, t‐tau, and p‐tau based on reproducibility across runs and have not changed over the time period of the current study. Types and counts of cells as well as concentrations of total protein and glucose in CSF were also noted. All participants or their legal representatives gave written consent for the lumbar puncture (LP) procedure.

### Statistical Analysis

Categorical variables were presented as count (%) and continuous variables as mean±SD or median (interquartile range), as appropriate based on their distribution. Categorical variables were analyzed using Fisher's exact test and continuous variables using the independent‐samples *t* test (for normal distributions) or Mann–Whitney *U* test (for nonnormal distributions). The relationship between 2 continuous variables was evaluated using bivariate correlation analyses. The relationship of CSF biomarker concentrations with imaging markers and clinical manifestations was assessed using univariate analysis and multivariate linear regression models after adjusting for age and sex. Analyses were repeated after excluding those with possible CAA or CAA‐related inflammation. As the goal of the analyses was to identify associations between CSF biomarkers with clinical and radiographic manifestations within patients with CAA, we did not include a healthy control group. Also, because the analyses were meant to be exploratory, they were not corrected for multiple comparisons, and results should be considered hypothesis generating rather than hypothesis confirming. Statistical analyses were performed using either Prism 10.1.1 (GraphPad) or SPSS (Statistical Package for Social Sciences for Mac version 24). Error bars represent SE of mean in the graphs. A *P* value <0.05 was considered statistically significant and all significance tests were 2 tailed.

## RESULTS

### Patient Characteristics

The study included 31 consecutive patients with CAA with adequate MRI sequences who underwent CSF testing for Aβ42, t‐tau, and p‐tau concentrations. Of these, 27 had probable CAA, 3 had possible CAA (strictly lobar intraparenchymal hemorrhage without other diagnostic hemorrhagic lesion or white matter features),^9^ and 1 had probable CAA with supporting pathology according to the Boston Criteria version 2.0.^9^ The mean age of patients was 69.6±8.4 years, and 19 (61.3%) were female. 20 (64.5%) presented with subacute to chronic subjective cognitive complaints, 9 (29%) with spontaneous acute or recent CAA‐related intracranial hemorrhage (ICH), and 2 (6.5%) with transient focal neurological episodes. Of the 20 who presented with cognitive complaints, 4 were found to have a prior lobar ICH on brain MRI. The median time between brain MRI and LP was 1 month (interquartile range, 0–5 months). Imaging markers of the study cohort are presented in the Table 1.

**Table 1:** Detailed Descriptives of Imaging Markers

Presence of intracerebral hemorrhage, n (%)	13 (41.9)
Lobar cerebral microbleed count, median (interquartile range)	20 (3–78)
Presence of cortical superficial siderosis, n (%)	16 (51.6)
Presence of lacunes (all lobar), n (%)	7 (22.6)
Enlarged perivascular space in centrum semiovale, n (%)	
Nonsevere (grade<3)	16 (51.6)
Severe (grade*≥*3)	15 (48.4)
Fazekas grade for white matter hyperintensities, n (%)	
Low degree (grade 1)	14 (45.2)
High degree (grade>1)	17 (54.8)

Twelve patients underwent LP for evaluation of cognitive complaints, 7 for evaluation for the presence of inflammation, and 12 to support a diagnosis of CAA to aid in clinical decision‐making, such as decisions regarding anticoagulation management in patients with history of other potential causes for microbleeds like cardiopulmonary bypass, traumatic brain injury, or infective endocarditis. Within the cohort, two patients were found to have lymphocytic pleocytosis on their CSF analyses concerning for CAA‐related inflammation, one of whom also underwent brain biopsy proving CAA‐related inflammation, and both were treated with corticosteroids.

Within our study cohort, the mean concentrations of CSF Aβ42 was 359.3±127 pg/mL, CSF t‐tau was 468±52 pg/mL and CSF p‐tau was 66.3±6.6 pg/mL. For comparison, previously reported CSF values obtained using the same ELISA assays for healthy individuals aged 61±8.7 years were 838±253 pg/mL for Aβ42, 215±78.3 pg/mL for t‐tau, and 47.9±14.8 pg/mL for p‐tau.^4^ Within our cohort, CSF Aβ42 concentrations did not correlate with CSF t‐tau or p‐tau concentrations (*P*=0.72 and *P*=0.32, respectively), but CSF t‐tau concentrations correlated with CSF p‐tau concentrations (r=0.79, *P*<0.001). Neither age nor sex was associated with the concentrations of any of the CSF biomarkers studied (*P*>0.1 for all comparisons).

### Relationship Between CSF Biomarkers and Clinical Presentations of CAA

**Figure 1:**
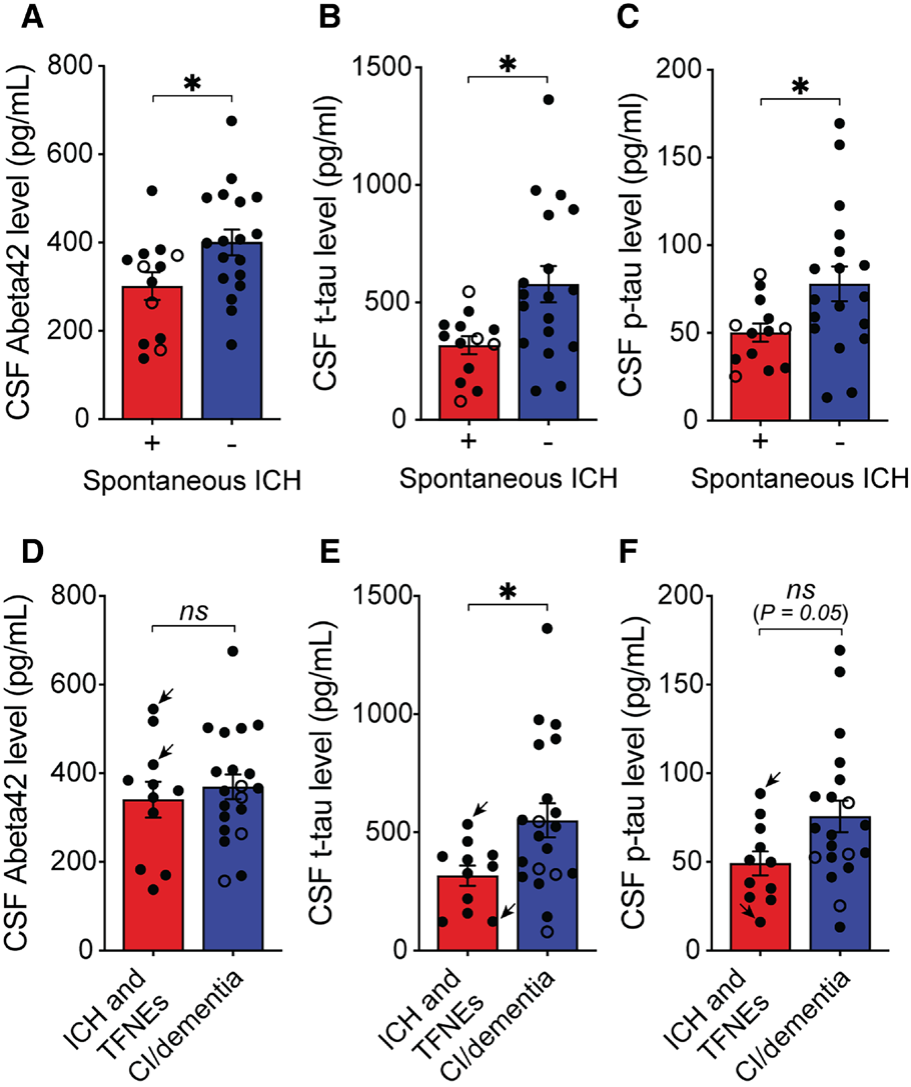
Comparison of Aβ42 (A), t‐tau (B), and p‐tau (C) concentrations between patients with CAA‐related intracranial hemorrhage and those without; and comparison of CSF Aβ42 (D), t‐tau (E), and p‐tau (F) concentrations between patients presenting with cognitive complaints and those without. The data points that belong to patients presenting with transient focal neurological episodes are marked with arrows (**D** through **F**), and data points that belong to patients presenting with cognitive complaints who were found to have prior ICH on imaging are shown with open circles (**A** through **F**). Comparisons were performed with 2‐tailed nonpaired *t* tests. * *P*<0.05. Aβ42 indicates amyloid‐beta 42; CAA, cerebral amyloid angiopathy; CI, cognitive impairment; CSF, cerebrospinal fluid; ICH, intracranial hemorrhage; ns, nonsignificance; p‐tau, phosphorylated tau at threonine 181; TFNE, transient focal neurological episode; and t‐tau, total tau.

Among the 13 patients with ICH on imaging, 11 had lobar intraparenchymal hemorrhage and 2 had convexity subarachnoid hemorrhage with angiography negative for aneurysms. The median time between the incident ICH and LP was 3 months (interquartile range, 1.25–23 months) and only 3 out of 13 patients had less than a month between the ICH and LP (6, 12, and 14 days respectively). We first compared CSF biomarker concentrations between patients with versus without CAA‐related ICH on imaging (Figure 1A through 1C). Patients who had ICH had lower CSF Aβ42 concentrations (ICH present 301.8±112 pg/mL versus ICH absent 400.9±123 pg/mL, *P*=0.029, Figure 1A) and lower concentrations of CSF t‐tau and p‐tau (t‐tau: ICH present 317.1±136 pg/mL versus ICH absent 577±326 pg/mL, *P*=0.006; p‐tau: ICH present 50.2±19.5 pg/mL versus ICH absent 77.9±42 pg/mL; *P*=0.020; Figure 1B and 1C), all of which remained significant in a linear regression model after adjusting for age and sex (*P*<0.05 for all comparisons). To rule out confounding effects of recent ICH on CSF biomarker concentrations, we excluded 3 patients with less than a month between ICH and LP. The difference in CSF Aβ42 concentrations became much more pronounced (ICH present 280.3±121 pg/mL versus ICH absent 400.9±123 pg/mL, *P*=0.019); whereas the differences in CSF t‐tau and p‐tau concentrations weakened, with the difference in p‐tau losing its statistical significance (t‐tau: ICH present 324.6±140 pg/mL versus ICH absent 577±326 pg/mL, *P*=0.029; p‐tau: ICH present 51.6±20 pg/mL versus ICH absent 77.9±42 pg/mL; *P*=0.075).

We ran the same analyses for patients with cognitive complaints (including 4 who also had imaging evidence of prior ICH) versus those without cognitive complaints. The mean age or sex did not differ between these groups (*P*>0.1 for all comparisons). The concentration of CSF t‐tau was higher in patients presented with cognitive complaints than in patients who did not have cognitive complaints (cognitive complaints 551±320 pg/mL versus others 317.2±141 pg/mL, *P*=0.03, Figure 1E). This association remained significant in a linear regression model after adjusting for age and sex (*P*=0.048). The concentration of p‐tau was also higher in patients with cognitive complaints than in patients without, just missing statistical significance (cognitive complaints 75.69±39 pg/mL versus others 49.24±22 pg/mL, *P*=0.052, Figure 1F), which remained nonsignificant in a linear regression model after adjusting for age and sex (*P*=0.082). Finally, CSF Aβ42 concentrations did not differ between patients with cognitive complaints and those without (cognitive complaints 369.5±125 pg/mL versus others 340.9±133 pg/mL, *P*=0.56, Figure 1D), which remained nonsignificant in a linear regression model after adjusting for age and sex (*P*>0.1).

### Association of CSF Biomarker Concentrations With Radiographic Manifestations of CAA

**Figure 2:**
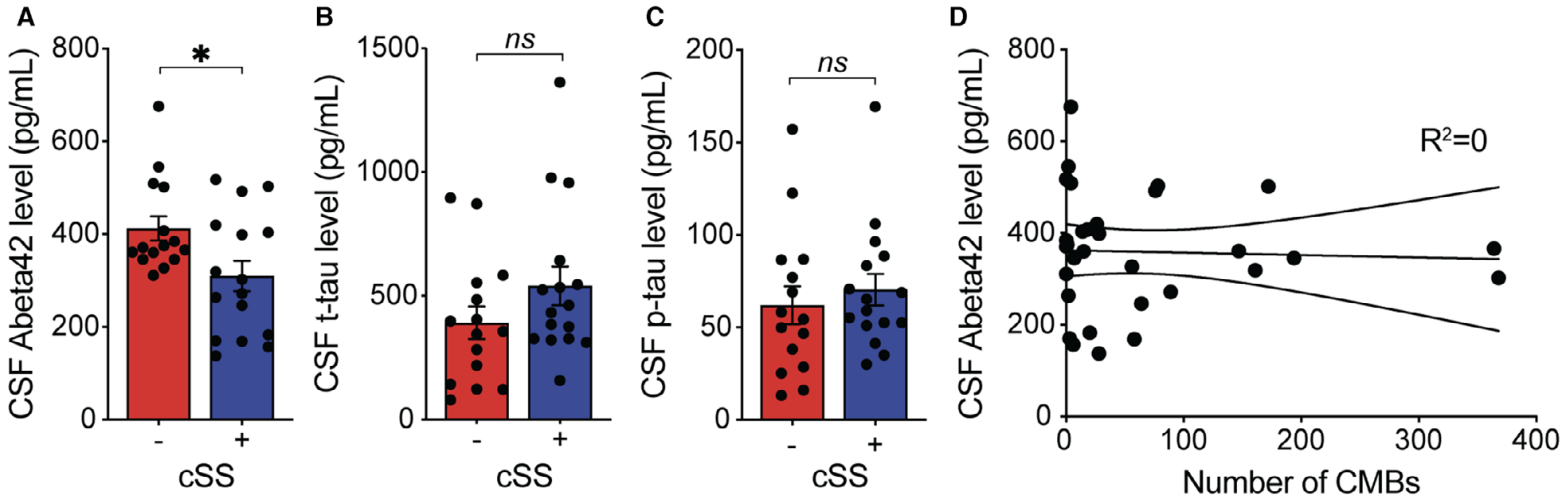
Comparison of Aβ42 (A), t‐tau (B), and p‐tau (C) between patients with cortical superficial siderosis and those without; **D, Absence of correlation between CSF Aβ42 concentrations and number of cortical microbleeds.** Comparisons were performed with 2‐tailed nonpaired *t* tests. * *P*<0.05. Aβ42 indicates amyloid‐beta 42; CMB, cerebral microbleed; CSF, cerebrospinal fluid; cSS, cortical superficial siderosis; ns, nonsignificance; p‐tau, phosphorylated tau at threonine 181; and t‐tau, total tau.

The concentration of CSF Aβ42 was lower in the 16 patients with at least 1 focus of cSS compared with the 15 without cSS (cSS present 309.6±131 mg/dL versus cSS absent 412.3±100 pg/mL, *P*=0.021, Figure 2A), which remained significant in a linear regression model after adjusting for age and sex (*P*=0.024). CSF t‐tau and p‐tau concentrations were not associated with the presence of cSS either in univariate analyses or regression models after adjusting for age and sex (*P*>0.1 for all comparisons, Figure 2B and 2C). To confirm that the significant decrease in CSF Aβ42 concentrations in those with cSS was indeed driven by the presence of cSS rather than co‐occurring ICH, we analyzed whether there was any association between presence of cSS and presence of ICH within our cohort. Among 16 patients with cSS, 6 (37.5%) had ICH and 10 (62.5%) did not have ICH; and there was no association between presence of cSS and presence of ICH (*P*=0.72, Fisher's exact test). A multiple regression model including presence of cSS and ICH for CSF Aβ42 concentrations showed independent contributions from both variables (*P*<0.01).

Lobar CMBs were present in 27 out of 31 patients, which limited our ability to reliably compare CSF biomarker concentrations between those with versus without CMBs. CSF Aβ42 concentrations did not correlate with the number of lobar CMBs (Figure 2D). CSF t‐tau and p‐tau concentrations also did not correlate with the number of lobar CMBs (*P*>0.1 for all comparisons, data not shown).

**Figure 3:**
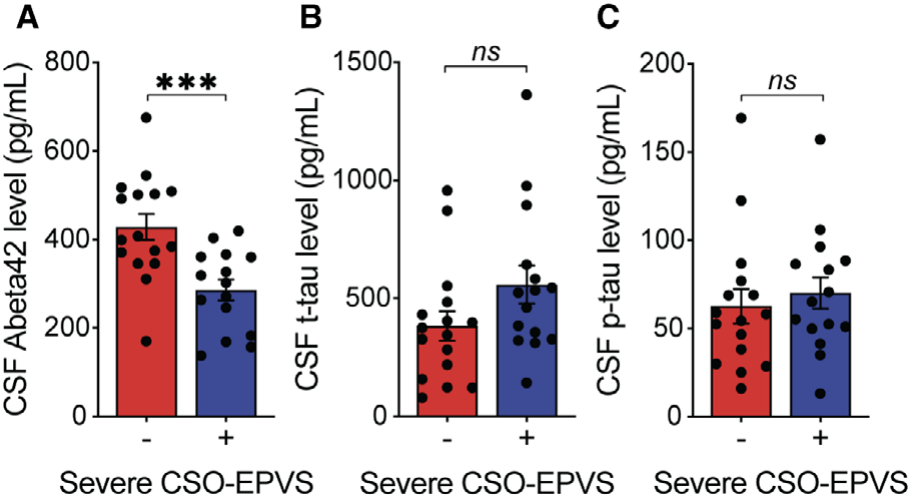
Comparison of Aβ42 (A), t‐tau (B), and p‐tau (C) between those with severe (grade 3 and 4) CSO‐EPVS and those without severe CSO‐EPVS (grade 1 and 2). *** *P*<0.001. Aβ42 indicates amyloid‐beta 42; CSF, cerebrospinal fluid; CSO‐EPVS, severe enlarged perivascular spaces in centrum semiovale; ns, nonsignificance; p‐tau, phosphorylated tau at threonine 181; and t‐tau, total tau.

The concentration of CSF Aβ42 was lower in the 15 patients with EPVS‐CSO as compared with the 16 without (severe EPVS‐CSO present 285.8±91 pg/mL versus severe EPVS‐CSO absent 428.3±117 pg/mL, *P*<0.001, Figure 3A). The concentrations of CSF t‐tau and p‐tau were not statistically different in between these 2 groups either in univariate or regression models after adjusting for age and sex (Figure 3B and 3C).

**Figure 4:**
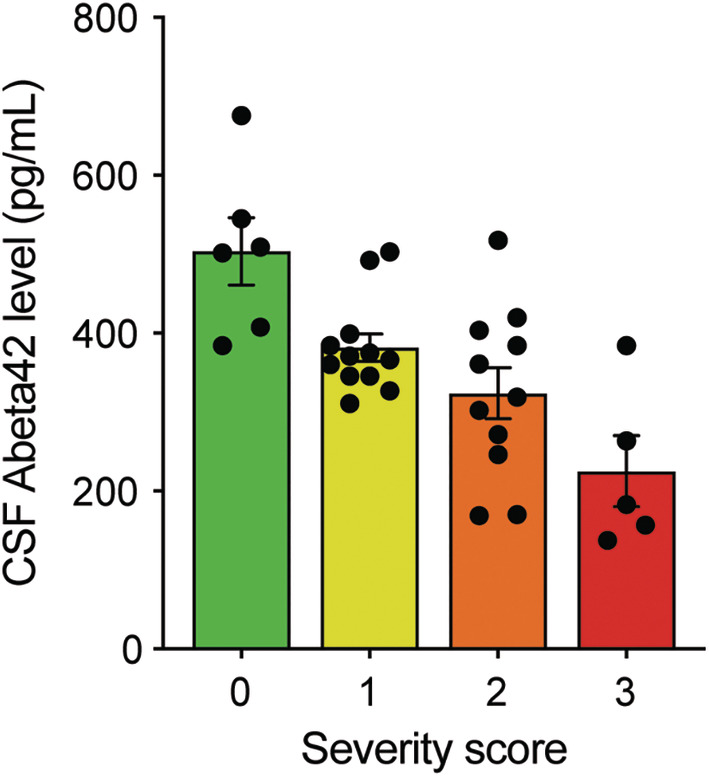
Aβ42 concentrations based on presence of CAA‐related macrobleeds (lobar intraparenchymal and convexity subarachnoid hemorrhage), cortical superficial siderosis, and enlarged perivascular spaces in centrum semiovale. We created an ordinal scoring system, where we assigned +1 for presence of each CSF Aβ42‐associated manifestation, thus 0 represents absence of all 3 features, whereas 3 represents presence of all 3 features. A significant negative correlation was observed (Spearman's *ρ*=−0.71, *P*<0.001), indicating an association between lower CSF Aβ42 concentrations and higher severity scores. Aβ42 indicates amyloid‐beta 42; CAA, cerebral amyloid angiopathy; CSF, cerebrospinal fluid; CSO‐EPVS, severe enlarged perivascular spaces in centrum semiovale; and ns, nonsignificance.

Because lower CSF Aβ42 concentrations were found to be associated with ICH, cSS, and severe EPVS‐CSO, a multiple regression model for CSF Aβ42 concentrations was run from these variables (F (3, 27)=14.227, *P*<0.001, R^2^=0.613). All variables but presence of cSS were independently associated with CSF Aβ42 (*P*<0.001). In order to further delineate whether lower CSF Aβ42 concentration is associated with more severe disease phenotype, we also created a post hoc ordinal scoring system, where we assigned a point for presence of each of the manifestations associated with lower CSF Aβ42. Here, a score of 0 represents absence of ICH, cSS and severe EPVS‐CSO, whereas a score of 3 represents presence of all 3 manifestations. Spearman's rank correlation analysis demonstrated a significant negative correlation between CSF Aβ42 levels and severity scores (ρ=−0.71, *P*<0.001), indicating that lower CSF Aβ42 levels are associated with higher severity scores (Figure 4).

In addition, the concentrations of CSF Aβ42, t‐tau, and p‐tau were not associated with the presence of lobar lacunes or WMH‐Fazekas grade (*P*>0.1 for all comparisons, data not shown). None of these results changed in regression models after adjusting for age and sex.

We repeated the analyses after excluding the 3 patients with possible CAA and 2 patients with CAA‐related inflammation (1 presumed based on presence of pleocytosis and 1 biopsy proven in addition to having pleocytosis). The findings of association between CSF biomarkers and cSS and severe CSO‐EPVS and the absence of association with lacunes and severe WMH remained statistically significant. The association between CSF Aβ42 and ICH became statistically marginal (*P*=0.06), likely due to slight reduction in sample size (as all 3 possible CAA cases presented with lobar ICH without other MRI manifestations of CAA). Out of 31 patients, 28 had susceptibility weighted imaging or susceptibility weighted angiographyand 3 had gradient echo as their T2*‐weighted susceptibility sequences. Because gradient echo is known to be less sensitive than susceptibility weighted imaging/susceptibility weighted angiography for detecting hemorrhagic lesions, we also repeated the analyses after excluding the 3 patients who had gradient echo and found similar results.

## DISCUSSION

In this study, we investigated the association between concentrations of 3 clinically testable and commercially available CSF biomarkers and the clinical and radiographic manifestations of CAA. Mean CSF concentrations of Aβ42, t‐tau, and p‐tau in this group of 31 patients diagnosed with CAA were very similar to those reported in a previous analysis of 17 patients assayed by a similar ELISA test in a research laboratory,^4^ supporting the reproducibility of the assay. In addition, compared with healthy controls from the same historical data set, those with CAA had lower Aβ42, higher t‐tau, and higher p‐tau,^4^ consistent with prior studies.^6^ We found that presence of spontaneous CAA‐related macrobleeds, cSS, and severe EPVS‐CSO were associated with lower CSF Aβ42 concentrations. Number of lobar CMBs, presence of lobar lacunes, and the severity of WMHs conversely were not associated with any of the CSF biomarker concentrations. Patients with CAA presenting with cognitive complaints had higher CSF concentrations of t‐tau and p‐tau. These findings suggest that the degree of vascular amyloid burden, as reflected by change in CSF Aβ42 concentrations, is differentially involved in the pathophysiology of different manifestations of CAA, whereas concentrations of tau species are most closely linked to cognitive complaints.

Cognitive complaints in those with CAA may, at least in part, be due to accompanying Alzheimer disease (AD) pathology. Patients with CAA who presented with subacute to chronic cognitive complaints had higher CSF t‐tau and p‐tau concentrations compared with those with noncognitive presentations such as ICH and transient focal neurological episodes (see Figure 1E and 1F). Because tau is considered a mediator of neurodegeneration in AD synergistic to amyloid,^16^, ^17^ higher tau concentrations among those with CAA might be indicative of the comorbid AD pathology, which is known to frequently co‐occur with CAA.^18^ Lower concentrations of CSF tau species were found in those who had CAA‐related ICH (Figure 1B and 1C), which is likely driven by the fact that almost all those who did not have ICH on imaging presented with cognitive complaints (Figure 1E and 1F).

Lower CSF Aβ42 concentration, reflecting higher vascular amyloid burden, was associated with CAA‐related macrobleeds. Prior amyloid PET studies have found higher amyloid signal in CAA patients compared with healthy controls and those with hypertensive deep ICH^19^ but have not systematically compared the degree of amyloid PET signal within a group of patients with CAA based on presence or absence of ICH. One study monitored 11 patients with CAA with serial brain MRIs after obtaining baseline amyloid‐PET with Pittsburgh compound‐B and showed that micro‐ and macrobleeds tend to occur within regions with higher Pittsburgh compound‐B signal; however, only 2 patients had macrobleeds whereas 9 patients had microbleeds, making it difficult to interpret the relationship of macrobleeds alone with amyloid burden.^20^ Here, we found that higher amyloid burden, as reflected by lower CSF Aβ42 concentrations, associated with presence of CAA‐related spontaneous ICH (lobar intraparenchymal hemorrhage and convexity subarachnoid hemorrhage) among patients with sporadic CAA. This association between higher amyloid burden and ICH is consistent with the finding in Dutch‐type hereditary CAA of higher amyloid PET signal and lower CSF amyloid concentrations in mutation carriers with symptomatic ICH compared with asymptomatic mutation carriers. Presence of cSS was also associated with lower CSF Aβ42 concentrations, consistent with the close link between symptomatic ICH and cSS established by previous studies.^11^, ^21^, ^22^, ^23^ The alternative explanation that CAA‐related ICH could cause lower CSF Aβ42 concentrations appears unlikely, as the majority of patients with CAA‐related ICH (10/13) underwent LP more than a month after the index bleed. When excluding the 3 patients who underwent LP within a month following the ICH from the analysis, the association between lower CSF Aβ42 concentrations and presence of ICH became more pronounced, suggesting that the observed reductions in Aβ42 were not merely a transient effect of recent hemorrhage but rather indicative of sustained amyloid burden. Of note, the minimum duration required for post‐ICH effects on CSF biomarker concentrations to dissipate, or whether there are any effects or not, is currently unknown, though we used 1 month as a cutoff here.

Despite the significant relationship between CSF Aβ42 concentrations and CAA‐related macrobleeds, the extend of microbleeds as measured by number of CMBs was not associated with CSF Aβ42 concentrations, suggesting that elements other than vascular amyloid burden might be at play. Supporting this result, a prior postmortem study of 12 patients with CAA also did not find a strong relationship between number of CMBs on ex vivo MRI and neuropathological CAA severity.^24^ Other candidate mechanisms for microbleeding include particular vessel characteristics such as wall thickness^25^ or perivascular inflammation.^26^

Among nonhemorrhagic manifestations, only the presence of severe EPVS‐CSO was found to be associated with lower CSF Aβ42 concentrations, which suggests that increased vascular amyloid burden is directly associated with appearance of CAA‐related EPVS. EPVS are hypothesized to arise via impaired perivascular clearance of solutes secondary to failures in vascular physiology from amyloid deposition within the vessel wall.^27^ A histopathological study of autopsied brains of those with CAA who underwent ex vivo MRI found that EPVS burden is locally associated with higher amyloid burden within the cortical vessels that give rise to the perforating cortical arterioles in the vicinity of EPVS.^27^ A similar association was also shown between higher burden of EPVS in CSO and increased amyloid PET signal.^28^, ^29^ Similar to that of ICH and cSS, degree of vascular amyloid burden seems to be directly involved in the pathophysiology of severe EPVS in CSO. We cannot exclude the possibility of a reverse relationship, where severe EPVS‐CSO reduces amyloid clearance into CSF leading to lower CSF Aβ42 concentrations.

Although we found that lower CSF Aβ42 concentrations were significantly associated with the presence of ICH, cSS, and severe EPVS‐CSO in univariate analyses, only ICH and severe EPVS‐CSO remained independently associated with lower Aβ42 concentrations in a multiple regression model, whereas the association with cSS lost its statistical significance. Interestingly, presence of ICH and cSS were not associated with each other and a multiple regression model including only these 2 parameters showed both were independently associated with lower CSF Aβ42 concentrations. This finding suggests that the initial association observed between cSS and lower Aβ42 concentrations in univariate analysis may have been confounded by the presence severe EPVS‐CSO. A prior study suggested that severe EPVS‐CSO was significantly related to cSS,^30^ which is further supported by our study, hinting toward a mechanistic link between severe EPVS‐CSO and cSS. In addition, although cSS lost its individual statistical significance in the multivariate model, our scoring system, which incorporates cSS along with ICH and EPVS‐CSO, still demonstrated a strong association with lower Aβ42 concentrations (see Figure 4), even if its individual association is less pronounced when adjusted for other factors. Furthermore, lower CSF Aβ42 concentrations with increasing number of CSF Aβ42‐associated manifestations of CAA in our scoring system suggests that CSF Aβ42 might be a potential biomarker for tracking the progression of symptomatic CAA. Because the diagnosis of probable CAA per Boston Criteria version 2.0 requires at least 1 hemorrhagic lesion, presymptomatic sporadic CAA (ie, before the first hemorrhagic lesion appears) cannot be detected in routine clinical care, limiting our ability of studying CSF Aβ42 in presymptomatic stage. However, we note that in Dutch‐type hereditary CAA, mutation carriers with symptomatic ICH have lower CSF Aβ42 concentrations than presymptomatic mutation carriers,^8^ supporting the possibility that CSF Aβ42 concentrations can also be useful in those with sporadic CAA at earlier disease stages.

We did not detect any change in Aβ42 or tau concentrations based on the severity of WMH scored by Fazekas grade. A prior amyloid‐PET study found that increased signal correlated with WMH volume in patients with CAA but not AD.^31^ This difference might be due to the higher rates of comorbid AD pathology in our cohort (more than half presented with subacute to chronic cognitive complaints), potentially weakening any association. Association with WMH might also be attenuated by factors other than CAA associated with this lesion, such as hypertension or other vascular risk factors. Lacunes also showed no association with the CSF biomarkers. In our cohort, those who had lacunes had strictly lobar lacunes, suggesting CAA over hypertensive small vessel disease as the most likely culprit.^32^ A previous study found that the majority of lobar lacunes were in contact with WMH and highly correlated with WMH severity,^32^ suggesting a possible common driver for both WMH and lobar lacunes that is distinct from the degree of vascular amyloid burden.

There are several limitations of this study that should be emphasized. First, the patients were selected from a cohort based on physician decision to obtain LP for biomarker testing, leading to a potential selection bias toward individuals requiring further data for clinical decision‐making as well as those presented with cognitive complaints. Second, although larger than many studies in this field, our sample size of 31 patients is relatively small, potentially leading to failure to detect some associations. In addition, the statistical analyses were not corrected for multiple comparisons as this study was meant to be exploratory and hypothesis generating rather than hypothesis confirming. For these reasons, it will be important to assess the generalizability and replicability of the current results in independent groups of patients with CAA with future studies. Finally, we note that the commercial CSF panel used in the current study did not measure Aβ40 concentrations, which might be a more accurate measure of severity of vascular amyloid deposition. Cerebrovascular amyloid in CAA is predominantly Aβ40 in CAA, contrasting with the predominance of Aβ42 in parenchymal plaques.^33^ Aβ42 accumulation within the vessel wall nonetheless appears to be an important component in CAA progression.^33^, ^34^ Furthermore, there have been robust data supporting reduced CSF Aβ42 concentrations correlating with vascular amyloid burden in CAA, including a population‐based autopsy study showing lower CSF Aβ42 concentrations in those with CAA compared with controls as well as in those with severe CAA compared with mild‐to‐moderate CAA based on histopathologically determined vascular amyloid burden, a relationship that continues to hold true even after removing participants with clinical dementia.^5^ In hereditary Dutch‐type CAA, where amyloid accumulation occurs almost exclusively within the vessel wall rather than brain parenchyma, lower CSF Aβ42 concentrations were detected, suggesting CSF Aβ42 is not only a marker of parenchymal accumulation but also a marker of vascular accumulation.^4^, ^8^ Finally, a recent CSF study found that decreased concentration of Aβ42 had the greatest area under the operator curve for differentiating sporadic CAA from healthy controls, and decreased concentration of Aβ40 had the greatest area under the operator curve for differentiating sporadic CAA from AD.^3^ An updated meta‐analysis also showed decreased CSF concentrations of both Aβ42 and Aβ40 in CAA compared with healthy controls.^35^ Even though CSF Aβ42 concentrations are lower in both CAA and AD,^35^ the CSF Aβ42–associated markers in our study, namely (1) lobar intraparenchymal hemorrhage and convexity subarachnoid hemorrhage, (2) cortical superficial siderosis, and (3) severe EPVS‐CSO, have been shown to be either the result of vascular amyloid deposition (CAA) or specifically associated with CAA pathology independent of parenchymal amyloid deposition (AD). As established by prior studies, hemorrhagic manifestations arise from vascular compromise secondary to amyloid deposition within vessel walls and thus would not be expected in pure AD without comorbid CAA.^1^, ^36^ Furthermore, severe EPVS‐CSO is specifically linked to CAA as evidenced by (1) its increased volume in Dutch‐type hereditary CAA,^37^ an essentially pure form of CAA with scant AD pathology; (2) the close pathological correlation between EPVS‐CSO and severe CAA in the cortex^27^; and (3) its independent association with higher lobar microbleed counts (a key hemorrhagic marker of vascular amyloid deposition) in cohorts with memory disorders.^38^, ^39^ Accordingly, although both CAA and AD can present with low CSF Aβ42, lower CSF Aβ42‐associated hemorrhagic markers and severe EPVS‐CSO highlight vascular amyloid pathology characteristic of CAA rather than AD. Although, similar analyses of CSF Aβ40 concentrations would be of interest, our results using a commercially available and accessible clinical assay for Aβ42, allows for potential translation of the results to clinical practice.

### Conclusions

In summary, we have found that lower CSF Aβ42 concentrations are associated with particular subtypes of brain injury in CAA, specifically CAA‐related macrobleeds (lobar intraparenchymal hemorrhage and convexity subarachnoid hemorrhage), cSS, and severe EPVS‐CSO. The results provide hints toward the mechanisms for these types of brain lesions and suggest CSF Aβ42 as a candidate biomarker for the clinical course and progression of CAA. These findings also offer some support for therapeutic reduction in vascular amyloid—via reduced production or increased clearance^40^—as plausible interventional approaches for lowering risk of CAA‐related ICH.

## Sources of Funding

Baris Alten was supported by Beverly Mahfuz Endowed Fund for Neurology Resident Training Sundry Fund.

## Disclosures

None.

## Supporting information

Figure S1

## References

[1] Greenberg SM , Bacskai BJ , Hernandez‐Guillamon M , Pruzin J , Sperling R , van Veluw SJ . Cerebral amyloid angiopathy and Alzheimer disease—one peptide, two pathways. Nat Rev Neurol. 2020;16:30–42. doi: 10.1038/s41582-019-0281-2 31827267 PMC7268202

[2] Koemans EA , Chhatwal JP , van Veluw SJ , van Etten ES , van Osch MJP , van Walderveen MAA , Sohrabi HR , Kozberg MG , Shirzadi Z , Terwindt GM , et al. Progression of cerebral amyloid angiopathy: a pathophysiological framework. Lancet Neurol. 2023;22:632–642. doi: 10.1016/S1474-4422(23)00114-X 37236210

[3] De Kort AM , Kuiperij HB , Marques TM , Jakel L , van den Berg E , Kersten I , van Berckel‐Smit HEP , Duering M , Stoops E , Abdo WF , et al. Decreased cerebrospinal fluid amyloid beta 38, 40, 42, and 43 levels in sporadic and hereditary cerebral amyloid angiopathy. Ann Neurol. 2023;93:1173–1186. doi: 10.1002/ana.26610 36707720 PMC10238617

[4] Verbeek MM , Kremer BP , Rikkert MO , Van Domburg PH , Skehan ME , Greenberg SM . Cerebrospinal fluid amyloid beta(40) is decreased in cerebral amyloid angiopathy. Ann Neurol. 2009;66:245–249. doi: 10.1002/ana.21694 19743453 PMC3697750

[5] Strozyk D , Blennow K , White LR , Launer LJ . CSF Abeta 42 levels correlate with amyloid‐neuropathology in a population‐based autopsy study. Neurology. 2003;60:652–656. doi: 10.1212/01.wnl.0000046581.81650.d0 12601108

[6] Charidimou A , Friedrich JO , Greenberg SM , Viswanathan A . Core cerebrospinal fluid biomarker profile in cerebral amyloid angiopathy: a meta‐analysis. Neurology. 2018;90:e754–e762. doi: 10.1212/WNL.0000000000005030 29386280 PMC5837868

[7] van Etten ES , Verbeek MM , van der Grond J , Zielman R , van Rooden S , van Zwet EW , Opstal AM , Haan J , Greenberg SM , van Buchem MA , et al. Beta‐amyloid in CSF: biomarker for preclinical cerebral amyloid angiopathy. Neurology. 2017;88:169–176. doi: 10.1212/WNL.0000000000003486 27903811 PMC5224714

[8] Schultz AP , Kloet RW , Sohrabi HR , van der Weerd L , van Rooden S , Wermer MJH , Moursel LG , Yaqub M , van Berckel BNM , Chatterjee P , et al. Amyloid imaging of dutch‐type hereditary cerebral amyloid angiopathy carriers. Ann Neurol. 2019;86:616–625. doi: 10.1002/ana.25560 31361916 PMC6876775

[9] Charidimou A , Boulouis G , Frosch MP , Baron JC , Pasi M , Albucher JF , Banerjee G , Barbato C , Bonneville F , Brandner S , et al. The Boston criteria version 2.0 for cerebral amyloid angiopathy: a multicentre, retrospective, MRI‐neuropathology diagnostic accuracy study. Lancet Neurol. 2022;21:714–725. doi: 10.1016/S1474-4422(22)00208-3 35841910 PMC9389452

[10] Greenberg SM , Vernooij MW , Cordonnier C , Viswanathan A , Al‐Shahi Salman R , Warach S , Launer LJ , Van Buchem MA , Breteler MM ; Microbleed Study G . Cerebral microbleeds: a guide to detection and interpretation. Lancet Neurol. 2009;8:165–174. doi: 10.1016/S1474-4422(09)70013-4 19161908 PMC3414436

[11] Charidimou A , Boulouis G , Roongpiboonsopit D , Auriel E , Pasi M , Haley K , van Etten ES , Martinez‐Ramirez S , Ayres A , Vashkevich A , et al. Cortical superficial siderosis multifocality in cerebral amyloid angiopathy: a prospective study. Neurology. 2017;89:2128–2135. doi: 10.1212/WNL.0000000000004665 29070669 PMC5696643

[12] Duering M , Biessels GJ , Brodtmann A , Chen C , Cordonnier C , de Leeuw FE , Debette S , Frayne R , Jouvent E , Rost NS , et al. Neuroimaging standards for research into small vessel disease‐advances since 2013. Lancet Neurol. 2023;22:602–618. doi: 10.1016/S1474-4422(23)00131-X 37236211

[13] Gokcal E , Horn MJ , van Veluw SJ , Frau‐Pascual A , Das AS , Pasi M , Fotiadis P , Warren AD , Schwab K , Rosand J , et al. Lacunes, microinfarcts, and vascular dysfunction in cerebral amyloid angiopathy. Neurology. 2021;96:e1646‐e1654. doi: 10.1212/WNL.0000000000011631 33536272 PMC8032369

[14] Charidimou A , Boulouis G , Pasi M , Auriel E , van Etten ES , Haley K , Ayres A , Schwab KM , Martinez‐Ramirez S , Goldstein JN , et al. MRI‐visible perivascular spaces in cerebral amyloid angiopathy and hypertensive arteriopathy. Neurology. 2017;88:1157–1164. doi: 10.1212/WNL.0000000000003746 28228568 PMC5373782

[15] Fazekas F , Chawluk JB , Alavi A , Hurtig HI , Zimmerman RA . MR signal abnormalities at 1.5 T in Alzheimer's dementia and normal aging. AJR Am J Roentgenol. 1987;149:351–356. doi: 10.2214/ajr.149.2.351 3496763

[16] Ballatore C , Lee VM , Trojanowski JQ . Tau‐mediated neurodegeneration in Alzheimer's disease and related disorders. Nat Rev Neurosci. 2007;8:663–672. doi: 10.1038/nrn2194 17684513

[17] Busche MA , Hyman BT . Synergy between amyloid‐beta and tau in Alzheimer's disease. Nat Neurosci. 2020;23:1183–1193. doi: 10.1038/s41593-020-0687-6 32778792 PMC11831977

[18] Brenowitz WD , Nelson PT , Besser LM , Heller KB , Kukull WA . Cerebral amyloid angiopathy and its co‐occurrence with Alzheimer's disease and other cerebrovascular neuropathologic changes. Neurobiol Aging. 2015;36:2702–2708. doi: 10.1016/j.neurobiolaging.2015.06.028 26239176 PMC4562901

[19] Farid K , Charidimou A , Baron JC . Amyloid positron emission tomography in sporadic cerebral amyloid angiopathy: a systematic critical update. Neuroimage Clin. 2017;15:247–263. doi: 10.1016/j.nicl.2017.05.002 28560150 PMC5435601

[20] Gurol ME , Dierksen G , Betensky R , Gidicsin C , Halpin A , Becker A , Carmasin J , Ayres A , Schwab K , Viswanathan A , et al. Predicting sites of new hemorrhage with amyloid imaging in cerebral amyloid angiopathy. Neurology. 2012;79:320–326. doi: 10.1212/WNL.0b013e31826043a9 22786597 PMC3400097

[21] Charidimou A , Boulouis G , Xiong L , Jessel MJ , Roongpiboonsopit D , Ayres A , Schwab KM , Rosand J , Gurol ME , Greenberg SM , et al. Cortical superficial siderosis and first‐ever cerebral hemorrhage in cerebral amyloid angiopathy. Neurology. 2017;88:1607–1614. doi: 10.1212/WNL.0000000000003866 28356458 PMC5405764

[22] Charidimou A , Boulouis G , Greenberg SM , Viswanathan A . Cortical superficial siderosis and bleeding risk in cerebral amyloid angiopathy: a meta‐analysis. Neurology. 2019;93:e2192–e2202. doi: 10.1212/WNL.0000000000008590 31732564 PMC6937489

[23] Roongpiboonsopit D , Charidimou A , William CM , Lauer A , Falcone GJ , Martinez‐Ramirez S , Biffi A , Ayres A , Vashkevich A , Awosika OO , et al. Cortical superficial siderosis predicts early recurrent lobar hemorrhage. Neurology. 2016;87:1863–1870. doi: 10.1212/WNL.0000000000003281 27694268 PMC5100711

[24] van Veluw SJ , Scherlek AA , Freeze WM , Ter Telgte A , van der Kouwe AJ , Bacskai BJ , Frosch MP , Greenberg SM . Different microvascular alterations underlie microbleeds and microinfarcts. Ann Neurol. 2019;86:279–292. doi: 10.1002/ana.25512 31152566 PMC8722100

[25] Greenberg SM , Nandigam RN , Delgado P , Betensky RA , Rosand J , Viswanathan A , Frosch MP , Smith EE . Microbleeds versus macrobleeds: evidence for distinct entities. Stroke. 2009;40:2382–2386. doi: 10.1161/STROKEAHA.109.548974 19443797 PMC2758289

[26] Kozberg MG , Yi I , Freeze WM , Auger CA , Scherlek AA , Greenberg SM , van Veluw SJ . Blood‐brain barrier leakage and perivascular inflammation in cerebral amyloid angiopathy. Brain Commun. 2022;4:fcac245. doi: 10.1093/braincomms/fcac245 36267331 PMC9576155

[27] Perosa V , Oltmer J , Munting LP , Freeze WM , Auger CA , Scherlek AA , van der Kouwe AJ , Iglesias JE , Atzeni A , Bacskai BJ , et al. Perivascular space dilation is associated with vascular amyloid‐beta accumulation in the overlying cortex. Acta Neuropathol. 2022;143:331–348. doi: 10.1007/s00401-021-02393-1 34928427 PMC9047512

[28] Charidimou A , Hong YT , Jager HR , Fox Z , Aigbirhio FI , Fryer TD , Menon DK , Warburton EA , Werring DJ , Baron JC . White matter perivascular spaces on magnetic resonance imaging: marker of cerebrovascular amyloid burden? Stroke. 2015;46:1707–1709. doi: 10.1161/STROKEAHA.115.009090 25908461

[29] Raposo N , Planton M , Payoux P , Peran P , Albucher JF , Calviere L , Viguier A , Rousseau V , Hitzel A , Chollet F , et al. Enlarged perivascular spaces and florbetapir uptake in patients with intracerebral hemorrhage. Eur J Nucl Med Mol Imaging. 2019;46:2339–2347. doi: 10.1007/s00259-019-04441-1 31359110

[30] Charidimou A , Jager RH , Peeters A , Vandermeeren Y , Laloux P , Baron JC , Werring DJ . White matter perivascular spaces are related to cortical superficial siderosis in cerebral amyloid angiopathy. Stroke. 2014;45:2930–2935. doi: 10.1161/STROKEAHA.114.005568 25116879

[31] Gurol ME , Viswanathan A , Gidicsin C , Hedden T , Martinez‐Ramirez S , Dumas A , Vashkevich A , Ayres AM , Auriel E , van Etten E , et al. Cerebral amyloid angiopathy burden associated with leukoaraiosis: a positron emission tomography/magnetic resonance imaging study. Ann Neurol. 2013;73:529–536. doi: 10.1002/ana.23830 23424091 PMC3715595

[32] Pasi M , Boulouis G , Fotiadis P , Auriel E , Charidimou A , Haley K , Ayres A , Schwab KM , Goldstein JN , Rosand J , et al. Distribution of lacunes in cerebral amyloid angiopathy and hypertensive small vessel disease. Neurology. 2017;88:2162–2168. doi: 10.1212/WNL.0000000000004007 28476760 PMC5467956

[33] Weller RO , Massey A , Newman TA , Hutchings M , Kuo YM , Roher AE . Cerebral amyloid angiopathy: amyloid beta accumulates in putative interstitial fluid drainage pathways in Alzheimer's disease. Am J Pathol. 1998;153:725–733. doi: 10.1016/s0002-9440(10)65616-7 9736023 PMC1853019

[34] Shinkai Y , Yoshimura M , Ito Y , Odaka A , Suzuki N , Yanagisawa K , Ihara Y . Amyloid beta‐proteins 1‐40 and 1‐42(43) in the soluble fraction of extra‐ and intracranial blood vessels. Ann Neurol. 1995;38:421–428. doi: 10.1002/ana.410380312 7668828

[35] Charidimou A , Boulouis G . Core CSF biomarker profile in cerebral amyloid angiopathy: updated meta‐analysis. Neurology. 2024;103:e209795. doi: 10.1212/WNL.0000000000209795 39270153

[36] Vernooij MW , Ikram MA , Hofman A , Krestin GP , Breteler MM , van der Lugt A . Superficial siderosis in the general population. Neurology. 2009;73:202–205. doi: 10.1212/WNL.0b013e3181ae7c5e 19620607

[37] Martinez‐Ramirez S , van Rooden S , Charidimou A , van Opstal AM , Wermer M , Gurol ME , Terwindt G , van der Grond J , Greenberg SM , van Buchem M , et al. Perivascular spaces volume in sporadic and hereditary (Dutch‐type) cerebral amyloid angiopathy. Stroke. 2018;49:1913–1919. doi: 10.1161/STROKEAHA.118.021137 30012821 PMC6202219

[38] Schrag M , McAuley G , Pomakian J , Jiffry A , Tung S , Mueller C , Vinters HV , Haacke EM , Holshouser B , Kido D , et al. Correlation of hypointensities in susceptibility‐weighted images to tissue histology in dementia patients with cerebral amyloid angiopathy: a postmortem MRI study. Acta Neuropathol. 2010;119:291–302. doi: 10.1007/s00401-009-0615-z 19937043 PMC2916065

[39] Martinez‐Ramirez S , Pontes‐Neto OM , Dumas AP , Auriel E , Halpin A , Quimby M , Gurol ME , Greenberg SM , Viswanathan A . Topography of dilated perivascular spaces in subjects from a memory clinic cohort. Neurology. 2013;80:1551–1556. doi: 10.1212/WNL.0b013e31828f1876 23553482 PMC3662325

[40] Zhang Y , Chen H , Li R , Sterling K , Song W . Amyloid beta‐based therapy for Alzheimer's disease: challenges, successes and future. Signal Transduct Target Ther. 2023;8:248. doi: 10.1038/s41392-023-01484-7 37386015 PMC10310781

