# Supplementary material for: Cerebrospinal Fluid Beta‐Amyloid Concentration and Clinical and Radiographic Manifestations of Cerebral Amyloid Angiopathy
Source: J Am Heart Assoc. 2025 Mar 3;14(5):e040025. doi: 10.1161/JAHA.124.040025 (PMC12132694; doi:10.1161/JAHA.124.040025)

SUPPLEMENTAL MATERIAL

Figure S1. Patient selection. A total of 52 unique patients were identified. Out of 52, 21 were excluded because of unavailability of CSF results, unavailability of adequate MRI images, or not meeting diagnostic criteria for CAA per Boston Criteria version 2.0. Of note, the results of ADMark assay were provided via a link from the source lab for 3 patients, which were no longer accessible at the time of chart review.

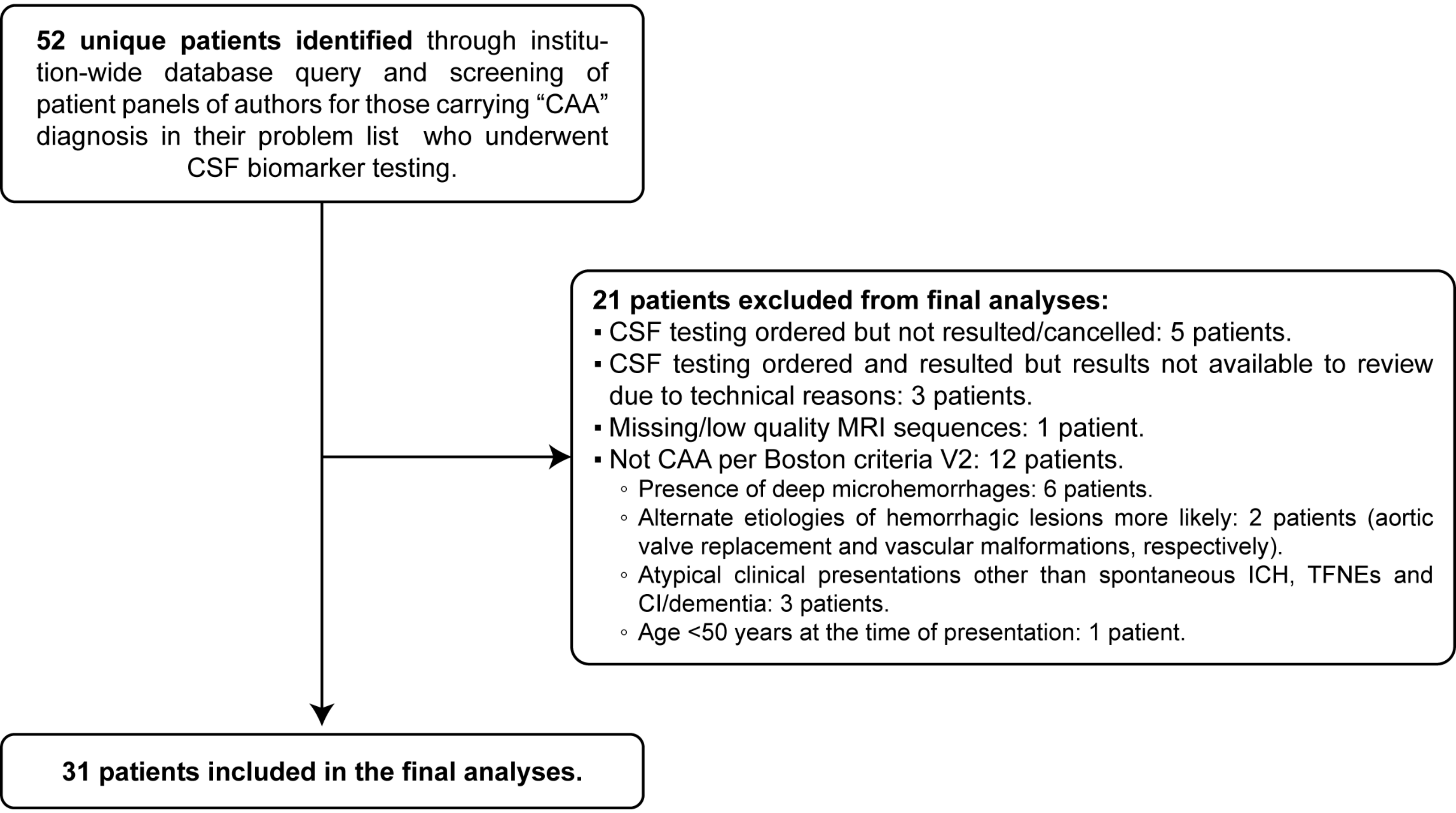

Supplement: Supplementary file 1 — Figure S1 [file JAH3-14-e040025-s001.pdf]
